# Application of Artificial Intelligence to the Diagnosis and Therapy of Nasopharyngeal Carcinoma

**DOI:** 10.3390/jcm12093077

**Published:** 2023-04-24

**Authors:** Xinggang Yang, Juan Wu, Xiyang Chen

**Affiliations:** 1 Division of Biotherapy, Cancer Center, State Key Laboratory of Biotherapy, West China Hospital, Sichuan University, Guoxue Road 37, Chengdu 610041, China; 2 Out-Patient Department, West China Hospital, Sichuan University, Guoxue Road 37, Chengdu 610041, China; 3 Division of Vascular Surgery, Department of General Surgery, West China Hospital, Sichuan University, Guoxue Road 37, Chengdu 610041, China

**Keywords:** artificial intelligence, nasopharyngeal carcinoma, nasopharyngoscopy, pathological biopsy, diagnosis, treatment, prognosis

## Abstract

Artificial intelligence (AI) is an interdisciplinary field that encompasses a wide range of computer science disciplines, including image recognition, machine learning, human−computer interaction, robotics and so on. Recently, AI, especially deep learning algorithms, has shown excellent performance in the field of image recognition, being able to automatically perform quantitative evaluation of complex medical image features to improve diagnostic accuracy and efficiency. AI has a wider and deeper application in the medical field of diagnosis, treatment and prognosis. Nasopharyngeal carcinoma (NPC) occurs frequently in southern China and Southeast Asian countries and is the most common head and neck cancer in the region. Detecting and treating NPC early is crucial for a good prognosis. This paper describes the basic concepts of AI, including traditional machine learning and deep learning algorithms, and their clinical applications of detecting and assessing NPC lesions, facilitating treatment and predicting prognosis. The main limitations of current AI technologies are briefly described, including interpretability issues, privacy and security and the need for large amounts of annotated data. Finally, we discuss the remaining challenges and the promising future of using AI to diagnose and treat NPC.

## 1. Introduction

Nasopharyngeal carcinoma (NPC), an epithelial carcinoma developing in the nasopharynx mucosal, is often observed at the pharyngeal recess [1]. Diagnosing NPC involves an endoscopy followed by an endoscopic biopsy of the suspected site [2,3]. Endoscopic biopsy may miss small cancers located submucosally or laterally to the pharyngeal crypt, which presents significant diagnostic challenges. Early diagnosis of NPC is difficult because of the late onset of symptoms and special anatomical structure. In most cases, NPC patients are diagnosed late, resulting in poor prognoses [4]. Local control rates have reached 95% in early NPC cases owing to the swift advancement of imaging techniques and radiotherapy [5]. Advanced-stage patients still have dismal outcomes, while advanced radiotherapy techniques and chemotherapy strategies have improved NPC prognosis [6,7]. Thus, it would be interesting to know if artificial intelligence (AI) can improve the diagnosis, therapy and prognosis prediction of NPC.

AI is a subdiscipline of computer science that recognizes the nature of intelligence and creates a new type of intelligent machine that can exhibit human-like behaviors [8]. AI is utilized in many areas, including medicine, communication, transportation and finance, among others [9]. AI is mainly used for disease diagnosis, treatment and prognosis prediction in the medicine area. Medical AI has two major branches: virtual and physical [10]. The virtual part of AI is composed of deep learning (DL) and machine learning (ML), which offer a potential way to construct robust computer-assisted approaches. The physical part of AI encompasses robots and medical devices [10]. Several recent studies have shown that AI can improve early diagnosis efficiency as well as the prognosis of NPC patients, through its application in diagnosis and treatment [11,12,13].

There are some reviews on the application of AI in NPC [13,14]. However, AI techniques are advancing so fast that it is necessary to update these reviews frequently. In this review, we analyze and summarize the research progress and clinical application of AI technologies in the diagnosis, treatment and prognosis prediction of NPC. We provide a complete picture of the current status of AI in the main clinical areas. We also study the state of the clinical implementation of AI and the effort needed to make progress in this area. We hope that this information will be helpful to both clinicians and researchers interested in the utilization of AI in the clinical care of NPC.

## 2. AI and Its Technologies

In the last decades, many medical imaging techniques have played a key role in the early detection, diagnosis and treatment of diseases, such as ultrasound, computed tomography (CT), magnetic resonance imaging (MRI) and positron emission computed tomography (PET-CT) [15]. Recently, significant advances have been made in AI, which allows machines to automatically analyze and interpret complex data [16]. AI is frequently used in some medical fields like oncology, radiology and pathology, which require accurate and plentiful image data analysis. Physicians usually detect, describe and monitor head and neck diseases by visually assessing head and neck medical images. This assessment is often based on experience and can be subjective. In contrast to qualitative reasoning, AI can make quantitative assessments by automatically recognizing imaging information [17]. AI, including traditional ML and DL, enables physicians to make more accurate and faster imaging diagnoses and greatly reduces workload.

Traditional ML algorithms are one of the AI approaches in medical imaging, which heavily rely on the pre-defined engineering features. These are defined by mathematical equations (e.g., tumor texture) and thus can be quantified using computer programs. Features are entered into ML models to help physicians classify patients and make clinical decisions. Traditional ML includes a large number of established methods, such as k-nearest neighbors (KNN), support vector machines (SVM), random forests (RF) and so on. These methods are widely used in radiology to convert image data into feature vectors through image processing methods. Predictive models are built by using these vectors to derive certain information from the same image data and then generating traditional ML. Radiomics have been evaluated in some small retrospective studies, which attempt to predict tissue subtypes, response to certain treatments, prognosis and other information from medical images of tumors.

DL, as a subset of ML, is based on a neural network structure inspired by the human brain. ML models must define and extract features from images and their performance depends on the quality of the features. In contrast, DL algorithms do not have to define features in advance [18]. They can automatically learn features and perform image classification and task processing. This data-driven model is more informative and practical. DL algorithms commonly used in medical image analysis and processing include the artificial neural network (ANN), deep neural network (DNN), convolutional neural network (CNN) and recurrent neural network (RNN). Currently, CNN is the most popular type of DL architecture in the field of medical image analysis [19]. The CNN consists of multiple layers, usually including convolutional, pooling and fully connected layers. The pixels in an image are aggregated and transformed by clustering through the convolutional layer to automatically extract high-level features. The deep convolutional neural network (DCNN) uses more convolutional layers and a larger parameter space to fit large-scale datasets. U-net uses full convolutional layers and image enhancement to obtain good accuracy with limited datasets. RNN is particularly unique in processing time series data. Different DL algorithms have different characteristics and application scenarios.

## 3. Screening of Studies

We performed a search using the following query: (“artificial intelligence” OR “machine learning” OR “deep learning”) AND (“nasopharyngeal carcinoma” OR “nasopharyngeal cancer”). Using the search phrase, a search of research articles from the past 15 years to March 2023 was performed on Springer, Google Scholar, PubMed and Embase. Because there are no indicators or validation protocols of consensus for the evaluation of each model’s performance, a holistic profile of this field was provided instead of a meta-analysis. From this perspective, loose inclusion and exclusion criteria were set (Table 1). Finally, a total of 76 studies were included after following the inclusion and exclusion criteria.

Only studies using AI techniques in NPC were selected. Table 1 shows the exclusion and inclusion criteria which were applied to papers based on the purpose of our review.

## 4. Applications of AI to NPC

**Figure 1:**
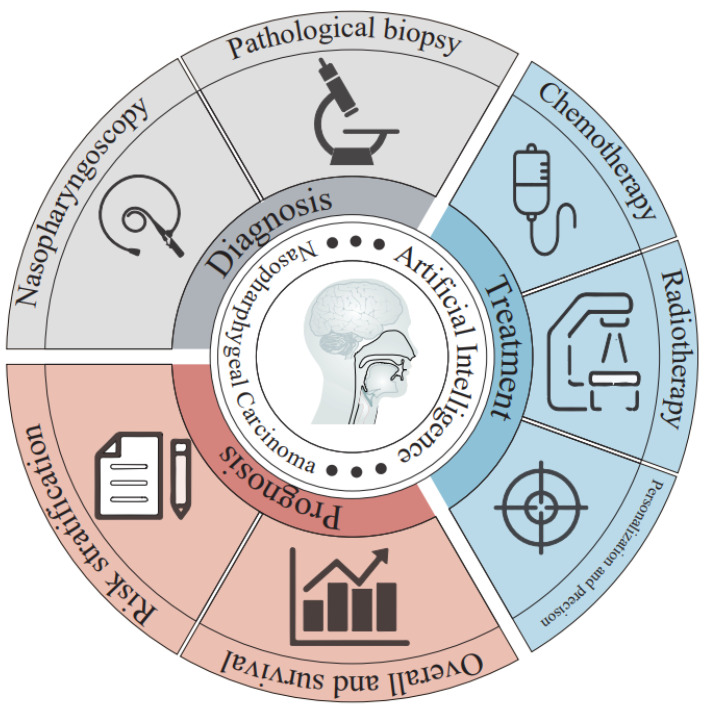
The application of AI in NPC diagnosis and treatment.

In the Lancet, a train of reviews entitled “Nasopharyngeal carcinoma” is published every few years [1,20,21,22]. In recent years, medical AI has been gaining popularity in the research of NPC. Many researchers have devoted themselves to NPC prediction of tumor detection, prognosis and efficacy of radiotherapy and chemotherapy (Figure 1).

### 4.1. AI and NPC Diagnosis

The diagnosis of NPC is a prerequisite for appropriate treatment, which can be divided into qualitative and staging diagnoses. Currently, qualitative diagnosis of NPC is dominated by the collection of biopsy tissue during endoscopy for pathological examination. Staging diagnosis mainly depends on imaging examinations, such as CT, MRI and PET-CT.

The fiberoptic nasopharyngoscope is a fiberoptic device that can magnify suspicious lesions up to thousands of times through the microscope’s visualization technique. The surgeon can use their own surgical forceps to biopsy the suspicious lesion tissue. The biopsy tissue is then selected and made into paraffin sections for histological examination under the microscope, with the help of electron microscopy or immunohistochemistry if necessary. CT scans a certain thickness of the human body with an X-ray beam, and the detector receives the X-rays passing through that layer. The converter converts the X-rays into digital signals, and the computer uses the digital signals to generate images. MRI uses the principle of nuclear magnetic resonance to detect the electromagnetic waves emitted by an applied gradient magnetic field. The magnetic field is based on the attenuation of the energy released in different structural environments within a substance, and can be used to map the internal structure of an object. PET-CT selectively reflects the metabolism of tissues and organs based on tracers, and the physiological, pathological, biochemical and metabolic changes of human tissues at the molecular level. At the same time, CT images are corrected for full energy attenuation of nuclear medicine images. Thus, the nuclear medicine images are able to completely achieve quantitative purposes and highly improve the accuracy of diagnosis, which realizes the complementary information of functional images and anatomical images.

It is difficult to perform accurate tumor diagnosis owing to the complexity of tumor symptoms and individual differences. AI technologies can help clinicians reduce their workload and improve the readability of imaging images, which leads to the improvement of accuracy and efficiency in diagnosing.

#### 4.1.1. AI Application in Nasopharyngoscopy

Nasopharyngoscopy allows direct observation of lesions on the nasopharyngeal wall, and physicians can analyze and screen lesion images to determine whether the lesions are associated with NPC. NPC diagnosis is currently done by visualizing suspicious tissue sites through using white-light reflectance endoscopy and taking biopsies. In previous studies, researchers developed different AI models using nasopharyngeal endoscopic images to distinguish NPC from nasopharyngeal benign hyperplasia. The studies showed that detection of NPC was not significantly different [23] or even performed better than that of radiologists [24]. In 2018, Mohammed et al. had three studies focusing on the detection of NPC using neural networks based on nasopharyngeal endoscopic images [25,26,27]. In all three studies, they used different neural network models and all achieved very good accuracy, sensitivity and specificity. Using 27,536 white-light imaging nasopharyngoscopy images, Li et al. developed a DL model for detecting NPC, reporting an accuracy of 88.7% and 88.0% on retrospective and prospective test sets, respectively [28].

However, conventional white-light endoscopy tends to miss superficial mucosal lesions. For this, Xu et al. designed and trained a Siamese DCNN, which can use white light and narrowband imaging images to enhance the performance of classification for the identification of NPC and non-carcinoma. They collected 4783 nasopharyngoscopy images for DL and validated the predictive power of the model for nasopharyngoscopy results. The overall accuracy and sensitivity of the model were 95.7% and 97.0% according to the prediction level of the patients [29].

Furthermore, the identification of normal tissues and treated NPC is a clinical challenge. For this reason, researchers developed a DL-based platform for fiber-optic Raman diagnostics. This platform utilizes multi-layer Raman-specific CNN. The optimized model can distinguish NPC from control and post-treatment patients with 82.09% diagnostic accuracy. The research team took a closer look at the saliency map of the best model. This map reveals specific Raman signatures associated with cancer-associated biomolecular variations [30].

#### 4.1.2. AI Application in Pathological Biopsy

A pathological biopsy in diagnosing NPC is required but remains challenging because of the non-keratinized carcinomas with little differentiation and many admixed lymphocytes in most samples. However, the diagnostic results of biopsy samples are often subjectively assessed by pathologists, which can lead to differences between observers. Diagnosing NPC by pathologists is ineffective and usually causes inconsistency in the results. Biopsy samples can be automatically classified and diagnosed by using AI techniques, which can improve diagnostic accuracy and efficiency, and reduce costs. The researchers trained and validated a DL model using 726 NPC biopsy specimens, reporting 0.9900 and 0.9848 areas under receiver operator characteristic curves (AUCs) at patch level and slide level, respectively [31]. Other researchers have also developed similar DL-dependent automated pathology diagnosis models. The model is based on the validation dataset and achieves an AUC of 0.869 for NPC diagnosis [32]. The outcomes indicate that the DL algorithm can recognize NPC and help pathologists improve their efficiency and accuracy.

In conclusion, AI plays an important role in recognizing and processing images, and in tissue segmentation in NPC (Table 2). While some applications of AI have yet to be fully realized, its potential in assisting NPC diagnosis is unquestionable.

### 4.2. AI and NPC Therapy

Major treatments for NPC include radiotherapy, chemotherapy and other integrated approaches. The application of AI techniques in NPC treatment can help clinicians design more personalized and accurate treatment plans for patients. The prediction of chemotherapy response and the precision of the radiotherapy process are usually combined with AI techniques in NPC therapy.

#### 4.2.1. AI Application in NPC Chemotherapy

Chemotherapy combined with radiotherapy is a great improvement in treating advanced NPC. Accurate pre-chemotherapy assessment can help NPC patients choose personalized treatment and improve their prognosis. In 2020, a research group developed a radiological map that integrates clinical data with radiomic features to predict the response and survival of NPC patients who received induced chemotherapy (IC). Based on survival analysis, IC responders had a significant advantage over non-responders in terms of progression-free survival [33]. In a study by Yang et al., CT texture analysis was used as a basis for developing a DL model to identify responders and non-responders to NPC IC. They extracted the DL features of the pre-trained CNN by a transfer learning method, and established the best performance model ResNet50 by SVM classification. The model demonstrated an AUC of 0.811 [34]. These models could be used to predict the treatment response to IC in locally advanced NPC, and might be a practical tool in deciding treatment strategies.

A pre-trained network is a saved CNN that has been previously trained on a large dataset. The original dataset is large and general enough that the spatial hierarchy learned by the pre-trained network can be used as an effective model for extracting features from the visual world. Even if the new problem and task are different from the original task, the learned features are portable between problems, which is an important advantage of DL. It makes DL very effective for small data problems.

To assess the effectiveness of DL on PET-CT-based radiomics for individual IC in advanced NPC, Peng et al. created radiomic signatures and nomograms. Based on a nomogram imaging analysis, high-risk and low-risk patients were divided into two groups, with high-risk patients benefiting from IC and low-risk patients not. Using it as a management tool for advanced NPC in the future would be a novel and helpful innovation [35].

#### 4.2.2. AI Application in NPC Radiotherapy

Radiotherapy is an indispensable treatment for NPC, in which tumor target segmentation and dose calculation are particularly critical. However, the overall radiotherapy planning process is always affected by image quality and the heavy workload of contouring tumor targets. Researchers have applied AI to radiotherapy planning to address these issues.

Image quality is fundamental to the whole of radiotherapy planning. However, high-quality CT images are usually not available owing to machine limitations and avoidance of human radiation during radiotherapy. AI can be used to enhance image quality. Tomotherapy uses megavoltage CT to verify the set-up and adapt radiotherapy, but its high noise and low contrast make the images inferior. In a study by Chen et al., synthetic kilovoltage CT was generated by using a DL approach. In the phantom study, synthetic kilovoltage CT showed significantly higher signal-to-noise ratio, image homogeneity and contrast ratio than megavoltage CT [36]. Li et al. used DCNN to generate synthetic CT images based on cone-beam CT and applied the images to dose calculation for NPC [37]. Similarly, Wang et al. applied DCNN to produce CT images based on T2-weighted MRI. Compared with real CT, most of the soft tissue and bone areas can be accurately reconstructed with synthetic CT [38]. Researchers developed an advanced DCNN architecture to generate synthetic CT images from MRI for intensity-modulated proton therapy treatment planning for NPC patients. The (3 mm/3%) gamma passing rates were above 97.32% for all synthetic CT images [39]. Through these methods, the image quality can be enhanced, which is conducive to tumor segmentation and dose calculation.

In addition, unimodal images are usually unable to provide enough information to accurately depict the tumor target region. As complementary information is provided by multiple form images, better radiotherapy treatment plans can be developed. In 2011, one study constructed a method utilizing weighted CT-MRI registration images for NPC delineation, called “SNAKE” [40]. Ma et al. developed a multi-modal segmentation structure using CNN, which is composed of multi-modal CNN and combined CNN for automatic NPC segmentation of CT and MR images [41]. Chen et al. developed a novel multi-modal MRI fusion network to accurately segment NPC [42]. Zhao et al. presented a method for automatically segmenting NPCs on dual-modality PET-CT images based on completely convolutional networks with auxiliary paths [43].

In current clinical practice, targets and organs-at-risk (OARs) are normally delineated manually by clinicians on CT images, which is tedious and time consuming. To address these issues, many automatic segmentation methods have been proposed by researchers. In one study, researchers proposed an adaptive thresholding technique based on self-organizing maps for semiautomated segmentation of NPC [44]. In addition, the team developed techniques based on region growing for segmentation of CT images for identifying NPC regions [45,46]. Bai et al. proposed an NPC-Seg DL algorithm for NPC segmentation using a location segmentation framework. In this study, the proposed algorithm was evaluated online on the StructseG-NPC dataset, and a 61.81% average dice similarity coefficient (DSC) was obtained on the test dataset [47]. Daoud et al. proposed a CNN model based on DL using a two-stage segmentation strategy to determine the final NPC segmentation by integrating three results obtained from coronal, axial and sagittal images. The study concluded that the DSCs of their proposed system were 0.87, 0.85 and 0.91 in the axial, coronal and sagittal profiles, respectively [48]. Li et al. created a DL model called U-net for NPC segmentation. After the training of the U-net model, the overall DSC of primary tumor was 74.00% [49]. In addition, many researchers have developed some improved models based on the U-net model to delineate the target volume of NPC. Through the training of the model, the final model obtained good DSC (0.827–0.84) [50,51,52]. Men et al. constructed an end-to-end deep deconvolutional neural network (DDNN) to segment nasopharyngeal gross tumor volume and clinical target volume. The performance of the DDNN and VGG-16 models are compared. The DSC values of DDNN were 80.9% of nasopharyngeal gross tumor volume and 82.6% of clinical target volume, while the DSC values of VGG-16 were 72.3% and 73.7%, respectively [53].

MRI images provide better soft tissue contrast compared with CT images, which facilitates accurate segmentation of the tumor target. There have been many studies on building various algorithms for NPC segmentation on MRI images. NPC contours were determined from MRI images using the nearest neighbor graph model and distance regularized level set evolution [54,55]. Li et al. utilized CNN to create an automatic NPC segmentation model based on enhanced MRI, and the trained model obtained a DSC of 0.89 [56]. Lin et al. built a 3D CNN architecture based on VoxResNet to automatically draw primary gross tumor volume profiles. In this study, 1021 NPCs were included and the trained model achieved a DSC of 0.79 [57]. Researchers developed a 3D CNN with long-range jump connections and multi-scale feature pyramids for NPC segmentation. The model has been trained and achieved a DSC of 0.737 in the tests [58]. Ye et al. successfully developed a fully automatic NPC segmentation method using dense connectivity embedding U-net and dual-sequence MRI images, with an average DSC of 0.87 in seven external subjects with NPC [59]. Luo et al. proposed the augmentation-invariant Strategy and combined it with the DL model. The final experimental results show that the augmentation-invariant Strategy is superior to the widely used nnU-net, which can perform highly accurate gross tumor volume segmentation on MRI for NPC [60].

NPC is highly malignant and invasive. Therefore, it is difficult to distinguish the boundaries between tumor tissue and normal tissue in a complex MRI context. In order to solve this background problem, researchers developed a coarse-to-fine deep neural network. The model firstly predicts the coarse mask based on the well-designed segmentation module, and then the boundary rendering module, which uses the semantic information from different feature mapping layers to refine the boundary of the coarse mask. The dataset encompassed 2000 MRI sections from 596 patients, and the model had a DSC of 0.703 [61].

CNN shows promising prospects for cancer segmentation on contrast-enhanced MRI, but some patients are not suitable for the use of contrast media. To address this issue, Wong et al. used U-net to delineate the primary NPC on non-contrast augmented MRI and compared it to the contrast-enhanced MRI. U-net showed similar performance (DSC = 0.71) of fat suppressant (FS)-T2W as enhanced -T1W, and CNN showed promise in depicting NPCs on FS-T2W images when contrast injection was desired [62].

Automated and precise segmentation of OAR can lead to more precise radiotherapy planning and reduce the risk of radioactive side effects. Researchers created a risk organ detection and segmentation network based on DL, and the DSCs of high-risk organ segmentation on CT images ranged from 0.689 to 0.934 [63]. Zhong et al. proposed a cascade network structure combining DL and the Boosting algorithm for segmentation of the organs-at-risk involving parotid gland, thyroid gland and optic nerve, with corresponding DSCs of 0.92, 0.92 and 0.89, respectively [64]. Peng et al. designed OrganNet, an improved full convolutional neural network for automatic segmentation of OARs, with an average DSC of 83.75% [65]. Zhao et al. designed an AU-net model based on 3D U-net to automatically segment the OARs of NPC and obtained a mean DSC value of 0.86 ± 0.02 [66].

The determination of radiotherapy dose also plays an important role in radiotherapy planning. Researchers developed a gated recurrent unit-based RNN model based on dosimetric information to predict treatment plans for NPC. An improved method is proposed to further improve the dose-volume histogram (DVH) prediction precision and the feasibility of this method for small sample patient data [67]. It is shown that the regenerated experimental plans (EPs) guided by the gated recurrent unit-based RNN prediction model achieve good agreement with the clinical plans (CPs). EPs save better doses for many OARs while still meeting acceptable criteria for planning tumor volume (PTV) [68,69]. Yue et al. developed a DL method for dose prediction of radiotherapy for NPC based on distance information and mask information. The predicted dose error and DVH error of the method were 7.51% and 11.6% lower, respectively, than those of the mask-based method [70]. Sun et al. developed a DL network based on U-net to predict the dose distribution of patients based on the anatomical structure information of patients. A total of 117 NPC cases were included in this study, which showed better organ retention and suboptimal planning target volume coverage using the voxel strategy [71]. Jiao et al. developed a generalized regression neural network using geometric and dosimetric information to predict OAR DVHs. The results showed that the R^2^ value increased by ~6.7% and the mean absolute error value decreased by ~46.7% after adding the dosimetric information to the DVH prediction [72]. Similarly, Chen et al. designed a CNN -based network based on a DL approach to directly predict the DVHs of OARs. The predicted differences between D2% and D50 can be controlled to within 2.32 and 0.69 Gy [73].

Some patients with NPC will develop complications after radiotherapy, which can affect the quality of life and lifespan. However, early diagnosis of the complications is a challenge. AI can be applied to the initial prediction of possible complications after NPC radiotherapy. Previous research used the random forest model to construct a radiological model for the early detection of radiation-induced temporal lobe injury (RTLI). In this model, RTLI can be dynamically predicted in advance, allowing early detection and the possibility of taking preventive measures to limit its progression [74]. Similarly, Bin et al. extracted radiological features from MRI and built a ML model to generate features. A nomogram integrating clinical factors was used to predict RTLI within 5 years after radiotherapy in patients with T4/N0-3/M0 NPC. The C-index of the validation cohort was 0.82 [75]. Ren et al. developed a prediction model based on a ML algorithm with dosimetric features. The model outperforms conventional dose-volume factors in predicting possible radiation-induced hypothyroidism in NPC patients receiving radiotherapy early and taking preventive measures for NPC patients. For prediction performance, the dosiomics-based prediction model showed better results at the optimal AUC value of 0.7, while the dose-volume factor-based prediction model showed better results at 0.61 [76]. To predict radiation-induced xerostomia, Chao et al. developed a clustering model that included inhomogeneous dose distributions within the parotid gland. The team combined clustering models with ML techniques to provide a promising tool for predicting xerostomia in head-and-neck-cancer patients [77].

#### 4.2.3. AI Application in the Personalized and Precise Treatment of NPC

Personalized and precise cancer treatment has become a major topic in NPC. Patients with locally advanced NPC can choose concurrent chemotherapy (CCRT) or IC plus CCRT as treatment options. However, their choice remains ambiguous. A DL-based NPC treatment decision model developed by researchers can predict the prognosis of patients with T3N1M0 NPC under different therapy regimens and recommend the optimized therapy accordingly. It is expected to be a potential tool to promote the individualized treatment of NPC [78]. The ability to discriminate between the different risks associated with NPC relapse in patients and to tailor individual treatment has become increasingly important. An AI model designed by researchers can divide relapse patients into different risk groups, which has great guidance potential for personalized treatment [79]. Targeted therapy is also important in treating NPC patients. Researchers developed a mathematical algorithm using SVM to predict the prognosis of NPC with advanced localization. The algorithm integrated the expression levels of multiple tissue molecular biomarkers representing tumor-genesis signaling pathways and serological biomarkers associated with EBV. It may guide future targeted therapies targeting related signaling pathways [80]. Moreover, the application of AI in clinical management is not easy to ignore. Previous research developed an automatic ML scoring system based on MRI data, which surpassed the American Joint Committee on Cancer (AJCC) [81] TNM system in the prognosis of NPC. Using the new scoring system can help improve counseling and personalized management of patients with NPC and help them achieve better outcomes [82].

With the arrival of the big data era, NPC therapy will become more personalized and precise (Table 3). The development of AI can not only effectively relieve clinicians, but also provide more accurate and humane medical services to patients.

### 4.3. AI and NPC Prognosis Prediction

Although great progress has been made in NPC treatment, the long-term prognosis of NPC patients is still unsatisfactory. The traditional TNM/AJCC staging system fails to provide the expected prognostic effect and to predict patient progression. In contrast, AI can accurately predict cancer survival time and progression through processing data and analyzing important features.

MRI images and clinical data are frequently used by researchers to build predictive models for NPC prognosis. Zhong et al. established a radiomic nomogram to predict disease-free survival. In the test cohort, the C-index of radiomic nomogram was 0.788 [83]. Researchers used SVM to construct radiomic ML models to predict disease progression, the models had good performance [84,85]. Li et al. combined radiomics and ML to predict the recurrence of NPC after radiotherapy, compared the centralized typical algorithm and the results showed that ANN achieved the best prediction accuracy of 0.812 [86]. Qiang et al. developed a prognosis model based on 3D DenseNet to predict disease-free survival of patients with non-metastatic NPC. A total of 1636 NPC patients were enrolled in the study. The model divided patients into low- and high-risk groups according to the cut-off value of risk score. The results showed that the model could correctly differentiate the two groups of patients (hazard ratio = 0.62) [87]. Similarly, Du et al. developed a DCNN model to assess the risk of non-metastatic NPC patients. In the validation set of 3-year disease progression, the AUC of the model was 0.828 [88]. In addition, several researchers have constructed similar DL models for prognostic prediction and risk stratification of NPC, all of which have good performance [78,89,90]. For NPC patients, survival prediction is of utmost importance. Jing et al. developed an end-to-end multi-modality deep survival network (MDSN) to precisely predict the risk of tumor progression of NPC patients. The model is compared with four traditional popular survival methods. Finally, the established MDSN performs best with a C-index of 0.651 [91]. Chen et al. used ML to develop a survival model based on tumor burden characteristics and all clinical factors. The study enrolled 1643 patients. The C-indexes were 0.766 and 0.760 in the internal validation and external validation sets [92].

PET-CT has particular advantages in sensitivity, specificity and accuracy in NPC recurrence and distant metastases. Meng et al. proposed a model based on pretreatment PET-CT images that can be used both to predict survival and segment advanced NPC. They adopt a hard-sharing segmentation backbone to aid in the extraction of regional attributes associated with the primary tumors and lessen the influence of irrelevant background data. Additionally, they also adopt a cascaded survival network to take the prognostic information from primary tumors and further utilize the tumor data acquired from the segmentation backbone [93]. Gu et al. developed an end-to-end multi-modal DL-based radiomics model to extract deep features from pre-processed PET-CT images and predict the 5-year progression-free survival. The team also incorporated TNM staging into the model to further improve prognostic power. A total of 257 patients with advanced NPC were enrolled and divided into internal and external cohorts. The AUC of the internal and external cohorts were 0.842 and 0.823, respectively [94].

Pathological images can also be used to construct a prognostic model for AI. Researchers integrated MRI-based radiological features and DCNN models based on pathology images and clinical features of NPC patients to construct a multi-scale nomogram to predict failure-free survival of NPC patients. The results showed that the C-index of the internal and external trial cohorts were 0.828 and 0.834, respectively [95]. In a previous study, the software QuPath (version 0.1.3. Queen’s University) was used to extract pathological microscopic features of NPC patients and the neural network DeepSurv to analyze the pathological microscopic features (DSPMF). In studies, DSPMF has proven to be a reliable prognostic tool and may guide treatment decisions for NPC patients [96].

Other researchers have used RNA data to build AI prediction models. In NPC, some miRNAs have prognostic power. Chen et al. combined miRNA expression data from various profiling platforms and constructed a predictive model using 6-miRNAsignatures. According to the functional analysis, the six miRNAs are principally involved in oncogenic signaling pathways, virus infection pathways and B-cell expression [97]. A metastatic and highly invasive cancer, NPC exhibits different molecular profiles and clinical outcomes in terms of their clinical characteristics. Zhao et al. applied ML techniques to RNA-Seq data from NPC tumor biopsies to identify 13 significant genes between the recurrence/metastasis and non-recurrence/metastasis groups. A 4-mRNA signature was identified using these genes. It shows good predictive value for NPC. A positive prognostic value was found for this signature for NPC. Moreover, the 4-mRNA signature was related to the immune response as well as cell proliferation [98]. Zhang et al. used the deep network to predict the prognosis of NPC based on MRI and gene expression, and the AUC was 0.88 [99].

AI makes it possible to predict outcomes based on diverse factors prior to treatment, which is beneficial for the whole diagnosis and treatment process (Table 4). In the near future, AI techniques will help doctors make rational and personalized medical decisions, including accurate diagnoses, personalized treatment and prognosis assessment for NPC patients.

### 4.4. Current State-of-the-Art AI Algorithms for NPC Diagnosis and Treatment

**Figure 2:**
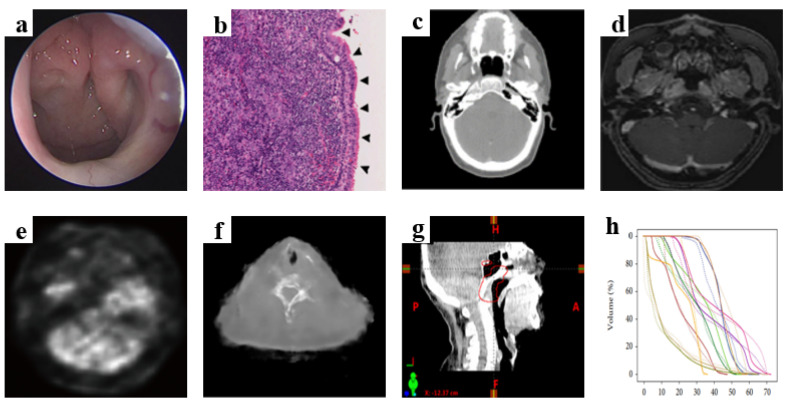
Sample images from various datasets: (**a**) endoscopic image (Mohammed et al., 2020 [26]); (**b**) whole slide image (Chuang et al., 2020 [31]); (**c**) CT image (Daoud et al., 2019 [48]); (**d**) MRI image (Guo et al., 2020 [58]); (**e**) PET image (Zhao et al., 2019 [43]); (**f**) CT-MR image (Wang et al., 2019 [38]); (**g**) CBCT-CT image (Li et al., 2019 [49]); (**h**) DVH image (Zhuang et al., 2021 [67]).

AI models require a large number of datasets for training and validation, and we have listed some sample images from various datasets in Figure 2.

AI can help doctors with statistics on pathology, physical examination reports, etc. It can analyze and mine patients’ medical data through technologies such as big data and deep mining to automatically identify patients’ clinical variables and indicators. A large part of the medical data comes from medical images, such as CT images, MRI images and PET-CT images. AI can help diagnose and treat diseases by learning a lot from medical images. CNNs have excellent performance in image recognition and image segmentation. In studies on diagnosis [28], treatment response prediction [33] and prognosis prediction [93] of NPC based on various images, researchers have obtained the best performance with improved models based on classical CNNs, usually using AUC and DSC as performance metrics. The FCN-based U-net model also shows very good performance for image segmentation, showing excellent performance in target segmentation [59] and dose prediction [69].

**Figure 3:**
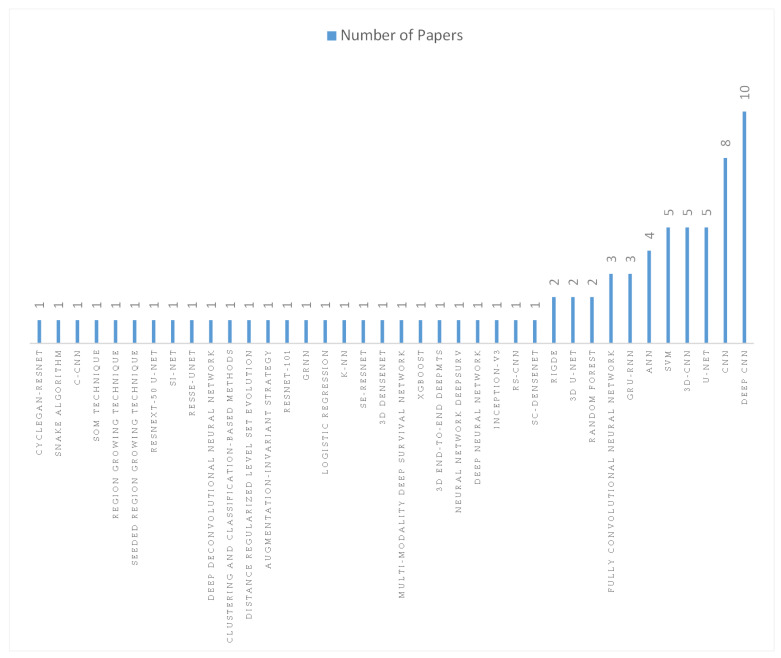
Artificial intelligence algorithms with the best performance in the papers included in our review.

The distribution of studies based on the best performing algorithms is shown in Figure 3. Many studies have improved on the classical model to create new algorithmic models. Among the AI algorithms, DCNN and CNN perform very well. However, the research results are based on each study independently and are not directly comparable due to the use of different datasets and/or evaluation metrics.

### 4.5. Common Training and Testing Methodologies

The performance of AI algorithms is influenced by many factors. We evaluated dataset size, class balance, validation strategy and data processing strategy, all of which have a direct impact on training and testing performance. A summary is given in Table 5.

Most of the research papers cited datasets with less than 1000 cases. In addition, only one study addressed and discussed the class balance. AI requires special strategies to manage limited and unbalanced data to reduce the impact on training and testing procedures (e.g., data augmentation techniques). Most studies use validation set and cross validation methods for model validation. The validation set method is the simplest method. It divides the entire data set into a training set and a test set. This method uses only a portion of the data for model training and is suitable for cases where the amount of data is relatively large. Cross validation uses the data repeatedly followed by slicing and dicing of the obtained sample data. We then combine the data into multiple different training and testing sets. This strategy is common in small datasets. The cross validation method will be repeated until each part is used as test data at least once. However, cross validation does not ensure the quality of ML models, as potentially biased or unbalanced data leads to biased evaluations. Some papers failed to describe any validation strategy.

Health data contain many missing values. AI algorithms are unable to handle missing values during data pre-processing, which leads to the deteriorated performance of the algorithms. According to Table 4, excluding cases with incomplete data is the most common strategy. However, this strategy suffers from significant information loss and performs poorly when missing values surpass the entire dataset. Some studies lack a data processing strategy and a detailed description of the management of the missing value cases. AI solutions are trained and tested on private/restricted datasets. These datasets either hold sensitive patient information, or belong to medical institutions that cannot or do not wish to make their data publicly available. Dataset availability improves reproducibility and transparency of research [100,101]. However, as all research papers used private data, the availability of datasets for AI applications in NPC remains a concern.

## 5. Current Challenges

Although there is rapid development of AI techniques in the clinical research of NPC, the application of AI remains immature [102]. Some challenges need to be addressed in order to translate these studies into clinically valuable applications.

As the survival period of NPC is prolonged, more and more patients are suffering from post-radiotherapy radiation brain injury, treatment failure and post-treatment recurrence and metastasis. These patients have complicated conditions and a poor prognosis, which has been causing hardships for treatment. To tackle the above mentioned problems, we need to find the economic, efficient and clinically optimal treatment plan for NPC. Because AI has the advantage of objectively analyzing and processing large amounts of data, AI is supposed to take part in establishing precise treatment ideas, including early screening, precise staging, precise target imaging, optimal treatment of recurrent metastatic NPC and the selection of combination treatment modalities. Prediction models constructed by AI algorithms require a large number of high-quality clinical data to improve their accuracy, sensitivity and specificity, so standardized data annotation and multicenter data sources are needed. Researchers have developed improved algorithms to handle small samples, with less accuracy [103]. At present, the AI algorithms of NPC are mostly limited to the data of a single medical institution [13]. It may lead to overfitting of the model, and the model is not fully applicable to a wider range of scenarios. Therefore, external validation is necessary before widespread clinical adaptation of AI applications.

In addition, AI predictions are called “black box” because the selection process and weighting process of AI algorithms are not clear. In other words, interpretability is an important consideration when applying AI to NPC. At present, there are two main solutions to this problem: interpretable models and model-independent interpretation methods [104]. Both approaches increase computational complexity. Therefore, much work remains to be done to improve the interpretability of the model.

Moreover, much of the research on the utilization of AI in NPC has been designed retrospectively. However, the encouraging results obtained in these studies need to be confirmed by further prospective and multicenter studies owing to possible selection bias in the retrospective study design.

Furthermore, privacy protection and data security are major challenges for AI. Building AI applications for NPC requires a large amount of clinical data from patients, requiring privacy protection and data security. Currently, there are no suitable technical solutions to alleviate this problem while meeting the growing demands of data-driven science [105]. Establishing a secure and reliable multicenter data sharing platform for the NPC is a possible way.

A common defect of current AI tools is their inability to deal with multi-tasking. No integrated AI system has been developed to detect multiple abnormalities in the human body. Disease and treatment require the use of multiple tools, in which the synergistic union is complicated. Leveraging AI solutions bring many benefits, while their deployment is difficult. For healthcare organizations, efforts are needed to bridge the skills gap by educating staff about AI systems and professional capabilities and building patient trust in AI.

## 6. Conclusions and Prospect

Literature reviews are broadly categorized as systematic and narrative. Systematic reviews are more rigorous in their methodology and less subject to bias than narrative reviews. However, the aim of this paper is to outline the dynamics of research advances in AI in the diagnosis and treatment of NPC and to present the challenges and future of the field. For this purpose, we have chosen to present a narrative review. To ensure the quality of the studies, we clarify the inclusion and exclusion criteria of the study, integrate and analyze the studies, pay attention to the shortcomings of the studied literature and ensure an objective evaluation attitude to give the reader a quick overview of the objective and comprehensive state of research in this field.

AI has shown great potential for applications in various clinical aspects of NPC, with the explosive growth of clinical data and research progress in ML and DL. The applications of AI to NPC are as follows: (1) understanding cancer at the molecular level through DL; (2) supporting the diagnosis and prognosis of NPC based on images and pathological specimens; (3) to promote personalized, accurate diagnosis and treatment of NPC. As AI techniques continue to advance, AI will have a great impact on the NPC clinical area. We believe that AI will be more closely combined with all aspects of medicine in the near future. We can rely on AI techniques to develop less invasive techniques than nasopharyngoscopy, with diagnostic accuracy close to that of pathological biopsies. We can build AI models based on clinical data to help healthy people understand early warning of NPC. AI will be closely integrated with radiotherapy to develop more personalized radiotherapy plans and conduct more effective whole-process efficacy evaluations. In the future, we can establish a large sample size and cross-population ethnic database to support the prediction of prognosis by AI techniques [106], to help researchers find the biggest prognostic factors and establish future prospective prognostic intervention studies.

## Figures and Tables

**Table 1:** Inclusion and exclusion criteria of the study.

Exclusion	Inclusion
Papers that were not written in English.	Journal articles published in the English language.
Full text of the document is not accessible on the internet.	Full-text papers that are accessible.
Relevant studies that are not based on deep learning or machine learning were used for modeling.	Machine learning algorithms were used for modeling.
The information of samples, the image data used, the modeling method or evaluation method are not described.	Deep learning algorithms were used for modeling.
Conferences papers, literature reviews and editorial materials that do not belong to original researchers.	The samples, the image data used, the modeling method and evaluation method are described in detail.

**Table 2:** Summary of AI models for NPC diagnosis session.

Author, Year	Purpose	Algorithms	Dataset	Best Algorithm	Best Algorithm Performance
Wong et al., 2021 [23]	NPC early detection	CNN	412 individuals	CNN	AUC: 0.960
Ke et sl., 2020 [24]	NPC detection and segmentation	SC-DenseNet	4100 individuals	SC-DenseNet	Accuracy: 0.978
Mohammed et al., 2018 [25]	NPC detection	ANN	381 endoscopic images	ANN	Accuracy: 0.962
Mohammed et al., 2020 [26]	NPC detection	ANN, region growing method	249 endoscopic images	ANN	Precision: 0.957
Abd Ghani et al., 2020 [27]	NPC detection	ANN, SVM, KNN	381 endoscopic images	ANN	Accuracy: 0.941
Li et al., 2018 [28]	NPC detection	fully convolutional network	27,536 biopsy-proven images of 7951 patients	fully convolutional network	Overall Accuracy: 0.887
Xu et al., 2022 [29]	NPC diagnosis	DCNN	4783 nasopharyngoscopy images of 671 patients	DCNN	AUC: 0.986
Shu et al., 2021 [30]	NPC diagnosis and post-treatment follow-up	RS-CNN	15,354 FP/HW in vivo Raman spectra of 418 subjects	RS-CNN	Overall accuracy: 0.821
Chuang et al., 2020 [31]	NPC identification	CNN	726 nasopharyngeal biopsies	CNN	AUC: 0.985
Diao et al., 2020 [32]	NPC identification	Inception-v3	1970 whole slide images of 731 cases	Inception-v3	AUC: 0.930

AI, artificial intelligence; NPC, nasopharyngeal carcinoma; CNN, convolutional neural network; AUC, area under the receiver operator characteristic curve; ANN, artificial neural network; SVM, support vector machines; KNN, k-nearest Neighbors; DCNN, deep convolutional neural network; FP, fingerprint; HW, high -wavenumber; RS-CNN, Raman-specified convolutional neural networks.

**Table 3:** Summary of AI applications for NPC treatment sessions.

Author, Year	Purpose	Algorithms	Dataset	Best Algorithm	Best Algorithm Performance
Zhao et al., 2020 [33]	IC treatment response and survival prediction	SVM	Multi-MR images of 123 patients	SVM	C-index: 0.863
Yang et al., 2022 [34]	IC treatment response prediction	CNN, Xception, VGG16, VGG19, InceptionV3, InceptionResNetV2	Medical records of 297 patients	CNN	AUC: 0.811
Peng et al., 2019 [35]	IC treatment response prediction	DCNN	PET-CT images of 707 patients	DCNN	C-index: 0.722
Chen et al., 2021 [36]	Synthetic CT generation and tumor segmentation	CycleGAN-Resnet, CycleGAN-Unet	Planning kV-CT and MV-CT images of 270 patients	CycleGAN-Resnet	Improvement: CNR 184.0% image uniformity 34.7% SNR 199.0% DSC: 0.790
Li et al., 2019 [37]	synthesized CT generation and dose calculations	DCNN	70 CBCT/CT paired images	DCNN	MAE: improved from (60, 120) to (6, 27) HU PTVnx70 1%/1 mm gamma pass rates: 98.6% ± 2.9%
Wang et al., 2019 [38]	synthetic CT generation	DCNN	CT/MRI images of 33 patients	DCNN	MAE: Soft tissue 97 ± 13 HU Bone 357 ± 44 HU
Chen et al., 2022 [39]	synthetic CT generation	DCNN	CT/MRI images of 206 patients	DCNN	The (3 mm/3%) gamma passing rates were above 97.32%
Fitton et al., 2011 [40]	NPC delineation	“Snake” algorithm	CT-MR images of 5 patients	“Snake” algorithm	Reducing the average delineation time by 6 min per case
Ma et al., 2019 [41]	NPC segmentation	C-CNN, M-CNN, S-CNN	CT-MR images of 90 patients	C-CNN	PPV: CT image 0.714 ± 0.089 MR image 0.797 ± 0.109
Chen et al., 2020 [42]	NPC segmentation	3D-CNN, U-net,3D U-net	MRI images of 149 patients	3D-CNN	DSC: 0.724
Zhao et al., 2019 [43]	NPC segmentation	Fully convolutional neural networks	PET-CT scans images of 30 patients	Fully convolutional neural networks	Mean dice score: 0.875
Weerayuth Chanapai et al., 2009 [44]	NPC Segmentation	SOM Technique	CT images of 131 patients	SOM Technique	CR: 0.620 PM: 0.730
Tatanun et al., 2010 [45]	NPC segmentation	Region growing technique	97 CT Images of 12 cases	Region growing technique	Accuracy: 0.951
Chanapai et al., 2012 [46]	NPC region segmentation	Seeded region growing technique	578 CT images of 31 patients	Seeded region growing technique	CR: 0.690 PM: 0.825
Bai et al., 2021 [47]	GTV segmentation	ResNeXt-50 U-net	CT images of 60 patients	ResNeXt-50 U-net	DSC: 0.618
Daoud et al., 2019 [48]	NPC segmentation	CNN, U-net	CT images of 70 patients	CNN	DSC: 0.910
Li et al., 2019 [49]	NPC Segmentation	U-net	CT images of 502 patients	U-net	DSC: 0.740
Xue et al., 2020 [50]	CTVp1 segmentation	SI-net	150 NPC patients	SI-net	DSC: 0.840
Jin et al., 2020 [51]	GTV Segmentation	ResSE-UNet	1757 annotated CT slices of 90 patients	ResSE-UNet	DSC: 0.840
Wang et al., 2020 [52]	GTV delineation	3D U-net, 3D CNN, 2D DDNN	CT images and corresponding manually delineated target of 205 patients	3D U-net	DSC: 0.827
Men et al., 2017 [53]	Target segmentation	DDNN, VGG-16	230 patients	DDNN	DSC: GTVnx 0.809 CTV 0.826
Huang et al., 2013 [54]	NPC Segmentation	Clustering and Classification-Based Methods with Learning, SVM	253 MRI slices	Clustering and Classification-Based Methods with Learning	PPV: 0.9345 Sensitivity: 0.9776
Huang et al., 2015 [55]	NPC segmentation	Distance regularized level set evolution	MR images of 26 patients	Distance regularized level set evolution	CR: 0.913 PM: 91.840
Li et al., 2018 [56]	NPC segmentation	CNN	MR images of 29 patients	CNN	DSC: 0.890
Lin et al., 2019 [57]	NPC segmentation	3D CNN	MR images of 1021 patients	3D CNN	DSC: 0.790
Guo et al., 2020 [58]	NPC segmentation	3D-CNN, 3D U-net, V-net, DDnet, DeepLab-like, CNN-based	MRI images of 120 patients	3D-CNN	DSC: 0.737
Ye et al., 2020 [59]	NPC segmentation	Dense connectivity embedding U-net	MRI images of 44 patients	Dense connectivity embedding U-net	DSC: 0.870
Luo et al., 2023 [60]	GTV delineation	augmentation-invariant Strategy, nnU-net	MRI images of 1057 patients	augmentation-invariant Strategy	DSC: 0.88
Li et al., 2020 [61]	NPC segmentation	ResNet-101, U-net, Attention U-net, BASNet, DANet, Unet++, RefineNet	2000 MRI slices of 596 patients	ResNet-101	DSC: 0.703
Wong et al., 2021 [62]	NPC delineation	U-net, CNN	non-contrast-enhanced MRI of 195 patients	U-net	DSC: 0.710
Liang et al., 2019 [63]	OARs detection and segmentation	CNN	CT images of 180 patients	CNN	DSC: 0.689–0.934
Zhong et al., 2019 [64]	OARs segmentation	Boosting-based cascaded CNN, FCN, U-net	CT images of 140 patients	Boosting-based cascaded CNN	DSC: Parotids 0.923 Thyroids 0.923 Optic nerves 0.893
Peng et al., 2023 [65]	OARs segmentation	fully convolutional neural network, U-net	CT images of 310 patients	fully convolutional neural network	DSC: 0.8375
Zhao et al., 2022 [66]	OARs segmentation	U-net	CT images of 147 patients	U-net	DSC: 0.86
Zhuang et al., 2021 [67]	DVH prediction	GRU-RNN	80 VMAT plans	GRU-RNN	coefficient r: EUD 0.976 Maximum dose 0.968
Cao et al., 2020 [68]	DVH prediction	GRU-RNN	100 VMAT plans	GRU-RNN	PTV70 CPs: 70.71 ± 0.83 EPs: 70.77 ± 0.28
Zhuang et al., 2019 [69]	DVH prediction	GRU-RNN	124 VMAT plans	GRU-RNN	PTV70 CPs: 70.90 ± 0.54 EPs: 71.40 ± 0.51
Yue et al., 2022 [70]	Dose prediction	3D U-net	Radiotherapy datasets of 161 patients	3D U-net	GTVnx 3 mm/3% gamma pass rate: 95.445%
Sun et al., 2022 [71]	Dose prediction	U-net	117 NPC patients	U-net	PTV70.4 D95(Gy): 70.4 ± 0.0
Jiao et al., 2019 [72]	DVH prediction	GRNN	106 nine-field IMRT plans	GRNN	Brainstem R^2^: 0.98 ± 0.02 Spinal cord R^2^: 0.98 ± 0.02
Chen et al., 2021 [73]	OARs DVHs prediction	CNN	180 cases	CNN	D2%(Gy): Brain stem PRV 0.06 ± 4.31 Spinal cord PRV −0.69 ± 1.77
Zhang et al., 2020 [74]	RTLI early detection	RF	MR images of 242 patients	RF	AUC: 0.83
Bin et al., 2022 [75]	RTLI prediction	SVM, RF	98 stage T4/N0e3/M0 patients	SVM	C-index: 0.82
Ren et al., 2021 [76]	Radiation-induced hypothyroidism prediction	LR, SVM, RF, KNN	145 patients	LR	AUC: 0.70
Chao et al., 2022 [77]	Radiotherapy-induced xerostomia prediction	SVM, KNN, RF	155 HNC patients	KNN	Mean accuracy: 0.68–0.7
Zhong et al., 2021 [78]	Treatment decision	SE-ResNet	MRI images of 638 stage T3N1M0 patients	SE-ResNet	HR: 0.17 and 6.24
Zhao et al., 2022 [79]	Risk stratification and survival prediction	light-weighted DCNN	PET-CT images and OS of 420 patients	light-weighted DCNN	C-index: 0.732
Jiang et al., 2016 [80]	Synchronous metastases NPC patients’ prognostic classifier	SVM	Hematological markers and clinical characteristics of 347 patients	SVM	HR: 3.45
Cui et al., 2020 [82]	NPC classification system	Rigde, Lasso	MR images of 792 patients	Rigde	AUC: OS 0.796 LRFS 0.721

AI, artificial intelligence; NPC, nasopharyngeal carcinoma; IC, Induction chemotherapy; MR, magnetic resonance; SVM, support vector machine; CNN, convolutional neural network; AUC, areas under receiver operator characteristic curve; PET-CT, positron emission tomography with computed tomography; DCNN, deep convolutional neural network; CT, computed tomography; kV-CT, kilovoltage computed tomography; MV-CT, megavoltage computed tomography; CNR, contrast-to-noise ratio; SNR, signal-to-noise ratio; DSC, dice similarity coefficient; CBCT, cone-beam computed tomography; MAE, mean absolute error; HU, Hounsfield units; PTVnx, 70 Gy to the planning target volume of the nasopharynx; MRI, magnetic resonance imaging; C-CNN, combined convolutional neural network; M-CNN, multi-modality convolutional neural network; S-CNN, single-modality convolutional neural network; PPV, positive predictive value; SOM, self-organizing map; CR, corresponding ratio; PM, perfect match; GTV, gross tumor volume; LR, logistic regression; KNN, k-nearest neighbor; CTVp1, primary tumor clinical target volume; SI-Net, sequential and iterative U-net; ResSE-UNet, Residual Squeeze-and-Excitation U-net; DDNN, deep deconvolutional neural network; CTV, clinical target volume; OARs, organs at risks; DVH, dose-volume histogram; VMAT, volumetric modulated arc therapy; GRU-RNN, gated recurrent unit-based recurrent neural network; EUD, equivalent uniform dose; CPs, clinical plans; EPs, experimental plans; IMRT, intensity-modulated radiation therapy; GRNN, generalized regression neural network; PRV, planning organ at risk volume; RTLI, radiation-induced temporal lobe injury; RF, random forest; HNC, head-and-neck-cancer; HR, hazard ratio; OS, overall survival; LRFS, local-region relapse-free survival.

**Table 4:** Summary of AI models for NPC prognosis session.

Author, Year	Purpose	Algorithms	Dataset	Best Algorithm	Best Algorithm Performance
Zhong et al., 2020 [83]	Survival prediction	DCNN	MRI images of 638 stage T3N1M0 patients	DCNN	C-index: 0.788
Zhuo et al., 2019 [84]	survival Stratification	SVM	658 non-metastatic patients	SVM	C-index: 0.814
Du et al., 2019 [85]	Early progression prediction	SVM	MRI images of 277 nonmetastatic patients	SVM	AUC: 0.80
Li et al., 2018 [86]	Recurrence prediction	ANN, KNN, SVM	306 patients	ANN	Accuracy: 0.812
Qiang et al., 2019 [87]	Disease-free survival prediction	3D DenseNet	MRI images of 1636 nonmetastatic patients	3D DenseNet	HR: 0.62
Du et al., 2019 [88]	Risk assessment	DCNN	MRI images of 596 nonmetastatic patients	DCNN	AUC: 0.828
Qiang et al., 2021 [89]	Risk stratification	3D CNN	MR images and clinical data of 3444 patients	3D CNN	C-index: 0.776
Zhang et al., 2021 [90]	DMFS prediction and treatment decision	DCNN	233 patients	DCNN	AUC: 0.796
Jing et al., 2020 [91]	Disease progression prediction	MDSN, BoostCI, LASSO -COX	Multi-parametric MRIs images of 1417 patients	MDSN	C-index: 0.651
Chen et al., 2021 [92]	Distant metastasis prediction	XGBoost	MRIs images of 1643 patients	XGBoost	C-index: 0.760
Meng et al., 2022 [93]	Joint survival prediction and tumor segmentation	3D end-to-end DeepMTS, LASSO-COX, DeepSurv, 2D CNN-based survival, 3D deep survival network	PET-CT images and clinical data of 193 patients	3D end-to-end DeepMTS	C-index: 0.722 DSC: 0.760
Gu et al., 2022 [94]	5-year PFS prediction	3D CNN	PET/CT images of 257 of patients	3D CNN	AUC: 0.823
Zhang et al., 2020 [95]	Prognosis prediction	DCNN	MRI images and biopsy specimens whole-slide images of 220 patients	DCNN	C-index: 0.834
Liu et al., 2020 [96]	Prognosis prediction	Neural network DeepSurv	H&E–stained slides of 1229 patients	Neural network DeepSurv	C-index: 0.723
Chen et al., 2021 [97]	Prognosis prediction	Ridge regression, elastic net	miRNA expression profiles and clinical data of 612 patients	Ridge regression, elastic net	5-year OS AUC: 0.70
Zhao et al., 2021 [98]	Prognosis prediction	RF	RNA-Seq data of 60 tumor biopsies	RF	AUC: OS 0.893 PFS 0.86.
Zhang et al., 2022 [99]	Prognosis prediction	DNN	MRI images and gene expression profiles of 151 patients	DNN	AUC: 0.88

AI, artificial intelligence; NPC, nasopharyngeal carcinoma; MRI, magnetic resonance imaging; DCNN, deep convolutional neural network; SVM, support vector machine; KNN, k-nearest neighbor; AUC, areas under receiver operator characteristic curve; HR, hazard ratio; CNN, convolutional neural network; DMFS, distant metastasis-free survival; MDSN, multi-modality deep survival network; PET-CT, positron emission tomography with computed tomography; DeepMTS, deep multi-task Survival; DSC, dice similarity coefficient; PFS, progression-free survival; H&E, Hematoxylin-eosin; OS, overall survival; RF, random forest; DNN, deep neural network.

**Table 5:** Summary of Training and Testing Methodologies.

Author, Year	Publicly Available	Balanced Classes	Validation Strategy	Data Handing Strategy
Wong et al., 2021 [23]	No	No	Cross validation	-
Ke et al., 2020 [24]	No	No	Validation set	Excluding
Mohammed et al., 2018 [25]	No	No	-	-
Mohammed et al., 2020 [26]	No	No	Cross validation	-
Abd Ghani et al., 2020 [27]	No	No	-	-
Li et al., 2018 [28]	No	No	Validation set	Excluding
Xu et al., 2022 [29]	No	No	Cross validation	-
Shu et al., 2021 [30]	No	No	Validation set	-
Chuang et al., 2020 [31]	No	No	Validation set	-
Diao et al., 2020 [32]	No	No	Validation set	-
Zhao et al., 2020 [33]	No	No	Validation set	Excluding
Yang et al., 2022 [34]	No	No	Validation set	Excluding
Peng et al., 2019 [35]	No	No	Validation set	Excluding
Chen et al., 2021 [36]	No	No	Validation set	Excluding
Li et al., 2019 [37]	No	No	Validation set	Excluding
Wang et al., 2019 [38]	No	No	Validation set	Excluding
Chen et al., 2022 [39]	No	No	-	Excluding
Fitton et al., 2011 [40]	No	No	-	-
Ma et al., 2019 [41]	No	No	-	Excluding
Chen et al., 2020 [42]	No	No	Cross validation	Excluding
Zhao et al., 2019 [43]	No	Yes	Cross validation	Excluding
Chanapai et al., 2009 [44]	No	No	Validation set	Excluding
Tatanun et al., 2010 [45]	No	No	-	Excluding
Chanapai et al., 2012 [46]	No	No	Validation set	Excluding
Bai et al., 2021 [47]	No	No	Cross validation	Excluding
Daoud et al., 2019 [48]	No	No	Cross validation	Excluding
Li et al., 2019 [49]	No	No	Validation set	Excluding
Xue et al., 2020 [50]	No	No	Validation set	Excluding
Jin et al., 2020 [51]	No	No	Validation set	Excluding
Wang et al., 2020 [52]	No	No	Cross validation	Excluding
Men et al., 2017 [53]	No	No	Validation set	Excluding
Huang et al., 2013 [54]	No	No	Validation set	Excluding
Huang et al., 2015 [55]	No	No	-	Excluding
Li et al., 2018 [56]	No	No	Cross validation	Excluding
Lin et al., 2019 [57]	No	No	Validation set	Excluding
Guo et al., 2020 [58]	No	No	Cross validation	Excluding
Ye et al., 2020 [59]	No	No	Cross validation	Excluding
Luo et al., 2023 [60]	No	No	Validation set	Excluding
Li et al., 2020 [61]	No	No	Validation set	Excluding
Wong et al., 2021 [62]	No	No	Cross validation	Excluding
Liang et al., 2019 [63]	No	No	-	Excluding
Zhong et al., 2019 [64]	No	No	Validation set	Excluding
Peng et al., 2023 [65]	No	No	Cross validation	Excluding
Zhao et al., 2022 [66]	No	No	Cross validation	Excluding
Zhuang et al., 2021 [67]	No	No	Validation set	Excluding
Cao et al., 2020 [68]	No	No	Validation set	Excluding
Zhuang et al., 2019 [69]	No	No	Validation set	Excluding
Yue et al., 2022 [70]	No	No	Validation set	Excluding
Sun et al., 2022 [71]	No	No	Validation set	Excluding
Jiao et al., 2019 [72]	No	No	Validation set	Excluding
Chen et al., 2021 [73]	No	No	Validation set	Excluding
Zhang et al., 2020 [74]	No	No	Validation set	Excluding
Bin et al., 2022 [75]	No	No	Cross validation	Excluding
Ren et al., 2021 [76]	No	No	Cross validation	Excluding
Chao et al., 2022 [77]	No	No	Cross validation	Excluding
Zhong et al., 2021 [78]	No	No	Validation set	Excluding
Zhao et al., 2022 [79]	No	No	Validation set	Excluding
Jiang et al., 2016 [80]	No	No	Validation set	Excluding
Cui et al., 2020 [82]	No	No	Cross validation	Excluding
Zhong et al., 2020 [83]	No	No	Cross validation	Excluding
Zhuo et al., 2019 [84]	No	No	Validation set	Excluding
Du et al., 2019 [85]	No	No	Validation set	Excluding
Li et al., 2018 [86]	No	No	Cross validation	Excluding
Qiang et al., 2019 [87]	No	No	Validation set	Excluding
Du et al., 2019 [88]	No	No	Validation set	Excluding
Qiang et al., 2021 [89]	No	No	Validation set	Excluding
Zhang et al., 2021 [90]	No	No	Validation set	Excluding
Jing et al., 2020 [91]	No	No	Validation set	Excluding
Chen et al., 2021 [92]	No	No	Validation set	Excluding
Meng et al., 2022 [93]	No	No	Cross validation	Excluding
Gu et al., 2022 [94]	No	No	Validation set	Excluding
Zhang et al., 2020 [95]	No	No	Validation set	Excluding
Liu et al., 2020 [96]	No	No	Validation set	Excluding
Chen et al., 2021 [97]	No	No	Validation set	Excluding
Zhao et al., 2021 [98]	No	No	Validation set	Excluding
Zhang et al., 2022 [99]	No	No	-	Excluding

## Data Availability

Data sharing is not applicable to this article.
